# Cadmium transport and tolerance in rice: perspectives for reducing grain cadmium accumulation

**DOI:** 10.1186/1939-8433-5-5

**Published:** 2012-02-27

**Authors:** Shimpei Uraguchi, Toru Fujiwara

**Affiliations:** 1 Graduate School of Agricunltural and Life Sciences, The University of Tokyo, Tokyo 113-8657, Japan

## Abstract

Cadmium (Cd) is a toxic heavy metal which harms human health. In Japan, a major source of human Cd-intake is rice grains and contamination of paddy soils by Cd and accumulation of Cd in rice grains are the serious agricultural issues. There also exist Cd contamination of rice and its toxicity in several populations in countries including China and Thailand. Understanding the Cd transport mechanisms in rice can be a basis for regulating rice Cd transport and accumulation by molecular engineering and marker-assisted breeding. Recently, a number of studies have revealed the behavior of Cd in rice, genetic diversity of Cd accumulation, quantitative trait loci controlling Cd accumulation and transporter molecules regulating Cd accumulation and distribution in rice. In this article, we summarize recent advances in the field and discuss perspectives to reduce grain Cd contents.

## Introduction

Cadmium (Cd) is a toxic heavy metal and is also known as one of the major environmental pollutants. Moderate Cd contamination of arable soils can result in considerable Cd accumulation in edible parts of crops (Arao and Ae 2003; Arao et al. 2003; Wolnik et al. 1983). Such levels of Cd in plants are not toxic to crops but can contribute to substantial Cd dietary intake by humans (Wagner 1993). In the case of "Itai-itai disease", Cd-polluted rice was the major source of Cd intake in the patients (Yamagata and Shigematsu 1970). This is the early case of chronic Cd toxicity in general populations without specific industrial exposure. Even in recent general populations in Japan, the internal Cd level is higher than those of other countries and this is largely because of daily consumption of Japanese rice which contains relatively high Cd (Watanabe et al., 1996; Watanabe et al. 2000; Tsukahara et al. 2003). Cd concentrations of recent Japanese rice have been constantly higher compared to those of other countries (Watanabe et al., 1996; Shimbo et al., 2001), although the values are much lower than the limit established by the Codex Alimentarius Commission of FAO/WHO (0.4 mg/kg). In some areas in China and Thailand, production of highly Cd-polluted rice and renal disfunctions among populations were reported (Nordberg et al., 1997; Jin et al., 2002; Honda et al., 2010). In the United States, increased consumption of rice and other cereals contributes to the recent increase of the dietary Cd intake (Egan et al. 2007). Many reports suggest importance to consider chronic effects of Cd exposure through foods (Jarup and Akesson 2009). In Japanese populations, the average dietary Cd intake (3.0 μg Cd/kg body weight/week) exceeds the tolerable weekly intake (2.5 μg Cd/kg body weight) set by the European Food Safety Authority (EFSA) and is about 50% of a provisional tolerable monthly intake (25 μg Cd/kg body weight/month) established by the Joint Food and Agriculture Organization/World Health Organization (FAO/WHO) Expert Committee on Food Additives and Contaminants (JECFA). Reeves and Chaney (2008) suggested to consider the high Cd availability of rice for humans because of relatively low iron and zinc contents in rice-based foods (Reeves and Chaney, 2008). These suggest the importance of reducing grain Cd accumulation in rice and other cereals for better human health.

Recently, as a model plant of cereals, physiological and molecular understanding of Cd transport in rice plants have been advanced. In this review, we describe current knowledge of rice Cd transporters and their (possible) roles in Cd accumulation. Several trials to generate "low-Cd-rice" based on these findings are also described.

### Physiology of rice Cd accumulation

The average Cd concentration in Japanese soils is 0.2 - 0.3 mg/kg DW (Takeda et al. 2004), which is somewhat higher than those of agricultural soils in China, Indonesia and the United States (Holmgren et al., 1993; Herawati et al., 2000). In paddy soils largely affected by industrial activities like mining and smelter, the Cd concentrations are much higher than the average (Xian, 1988; Asami et al., 1995). In agricultural soils, atmospheric deposition (Keller and Schulin, 2003) is known as a major source of Cd input. In paddy fields, irrigation water is another Cd source which continuously loads Cd into soils (Kikuchi et al. 2007).

Rice absorbs Cd^2+ ^in soils, and after several processes of transport Cd finally accumulates into grains. Cd is rapidly transported from roots to shoots by the xylem after absorption (Uraguchi et al. 2009b). Substantial Cd is detected in the xylem sap and shoot tissues 1 h after Cd treatment to roots, and this activity of root-to-shoot translocation by the xylem is the determinant for shoot Cd accumulation level. On the other hand, in the panicle neck, phloem is the major Cd transport route into grains (Tanaka et al. 2007). In phloem sap, Cd binds to an unknown 13 kDa protein and SH-compounds (Kato et al. 2010). The real-time live-imaging technique using a positron emitting radio isotopes called PETIS revealed the detailed behavior of Cd in rice after absorption (Fujimaki et al. 2010). They demonstrated that Cd is rapidly translocated from roots to shoots through culms and Cd tends to be retained in nodes. And after 7 h of Cd treatment, Cd is preferentially deposited into panicles rather than into leaf blades. These suggest that nodes are the important tissue for redirecting Cd transport from roots probably by transferring Cd from xylem to phloem. In addition to Cd absorbed from roots, remobilization of Cd in leaf blades is also likely to contribute to grain Cd accumulation (Rodda et al. 2011). They suggest that a substantial amount of Cd accumulated in leaf blades before heading is remobilized and transported into grains during the ripening stage.

**Figure 1:**
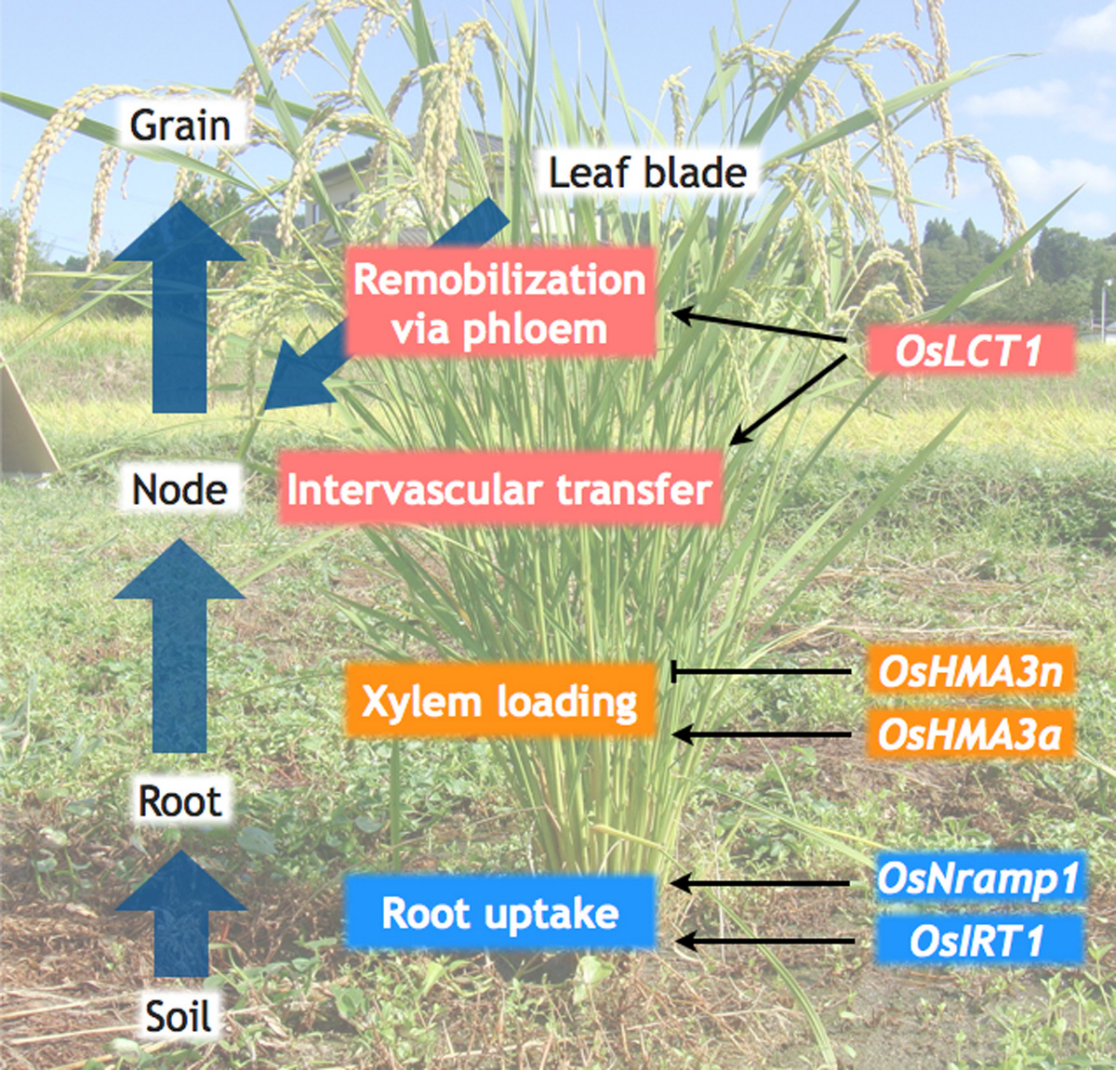
**A schematic model of Cd transport processes from soil to grains in rice**. Cd is absorbed from soils into roots. OsIRT1 and OsNramp1 are suggested to mediate this process. OsHMA3n (the functional allele of OsHMA3) play a critical role in Cd compartmentation into vacuoles in root cells and thus negatively regulates Cd xylem loading. OsHMA3a (the non-functional allele of OsHMA3) can not function in vacuolar Cd compartmentation in roots and which results in high efficiency of root-to-shoot Cd translocation. OsLCT1 contributes to Cd remobilization from leaf blades via phloem and also is likely to play a part in intervascular Cd transfer at nodes.

These physiological studies indicate four major transport processes for rice Cd accumulation: (1) root Cd uptake, (2) root-to-shoot translocation by xylem flow, (3) redirection at nodes and (4) remobilization from leaves (Figure 1). After the first report by Ishikawa et al. (2005), several studies conducted QTL analyses to identify the responsible transporter gene for these processes (Ishikawa et al. 2010; Ishikawa et al. 2005; Tezuka et al. 2010; Ueno et al. 2009). QTL analysis is a very useful approach because there is a clear genotypic difference in Cd accumulation in shoots and grains among cultivars. Generally, Cd accumulation in shoots and grains are potentially higher in *indica *cultivars compared to *japonica *cultivars (Arao and Ae 2003; He et al., 2006b; Uraguchi et al. 2009b) and moreover, some specific cultivars among *indica *rice accumulate much higher Cd in vegetative tissues and grains (Uraguchi et al. 2009b). Recently, several transporters have been identified as a Cd transporter in rice by forward and reverse genetics and their functions in these processes will be reviewed in the following sections and summarized in Figure 1.

### Uptake by roots

In plants and mammals, many of transporters for divalent transition metals have a Cd^2+ ^uptake activity. In mammals, ZIP8 and ZIP14, two transporters belonging to the Zinc-regulated transporter (ZRT) -like, Iron-regulated transporter (IRT) -like protein (ZIP) family, transport Cd^2+ ^as well as Mn^2+^, Fe^2+ ^and Zn^2+ ^(He et al. 2006a; Himeno et al. 2009; Nebert et al. 2009). In plants, it has been demonstrated much earlier than in mammals that AtIRT1, a ZIP family transporter for Fe^2+^, Zn^2+ ^and Mn^2+ ^also mediates Cd uptake in roots of *Arabidopsis thaliana *(Connolly et al. 2002; Vert et al. 2002). AtIRT1 is the primary transporter for the strategy-1 iron (Fe^2+^) uptake system in *A. thaliana*. In rice, iron is mainly absorbed by the strategy-2 system in the form of Fe-phytosiderophore (Romheld and Marschner 1986), but Fe^2+ ^transporters have been also identified which may function in Fe^2+ ^uptake by roots (Ishimaru et al. 2006). OsIRT1 and OsIRT2 have an influx activity of Cd^2+ ^as well as Fe^2+ ^in yeasts, suggesting that OsIRTs play some role in root Cd uptake especially after release of pounded water during intermittent water management (Ishimaru et al. 2006; Nakanishi et al. 2006). They suggested that under flooded paddy soils, *OsIRT*s might be induced by lower levels of available iron and after water release induced OsIRTs might contribute to uptake of Cd which was much available in aerobic conditions. When *OsIRT1 *was overexpressed, Cd accumulation in roots and shoots was increased under MS medium containing excess Cd, but this phenotype was not observed in the field condition (Lee and An 2009). These suggest that OsIRT1 is potentially involved in root Cd uptake but its contribution is largely affected by the environmental (soil) conditions.

Transporters of natural resistance-associated macrophage protein (Nramp) family are also known to mediate Cd transport. In mammals, DCT1/DMT1/Nramp2 functions in Cd^2+ ^uptake as well as uptake of Zn, Mn, Fe, Co and Ni ions (Gunshin et al. 1997; Hosoyamada et al. 2003). In plants, at the same time, it has been demonstrated that *AtNramp1*, *AtNramp3 *and *AtNramp4 *mediate inward Cd transport in yeasts and overexpression of *AtNramp3 *resulted in increased sensitivity to Cd (Thomine et al. 2000). In rice, there are seven *Nramp *genes. OsNramp1, an iron transporter, has been established as a Cd-influx transporter in the plasma-membrane (Takahashi et al. 2011). The cell-type specificity of *OsNramp1 *expression was not examined but the mRNA expression was much higher in roots than in shoots. *OsNramp1*-overexpressing rice plants accumulated less Cd in roots and much Cd in shoots compared to the wild-type plants when grown in media containing 1 μM or less Cd, suggesting that OsNramp1 is possibly involved in Cd transport into roots. Much interesting finding of their report is higher expression of *OsNramp1 *in *indica *cultivars. Comparing Sasanishiki (a *japonica *cultivar) and Habataki (a *indica *cultivar), the root-to-shoot Cd translocation ability is greater in Habataki (Uraguchi et al. 2009b). Following QTL analysis of a mapping population obtained by crossing the two cultivars, a major QTL for high Cd accumulation in shoots of Habataki was detected on the short arm of Chr. 7 (Ishikawa et al. 2010). Although the responsible gene has not been identified, this QTL contains *OsNramp1*. There was no difference in the amino acid sequences of OsNramp1 between Habataki and Sasanishiki, but the gene expression was higher in Habataki and other *indica *cultivars probably caused by several insertion and deletion in the promoter region (Takahashi et al. 2011). This higher expression of *OsNramp1 *in *indica *cultivars may partly explain higher Cd accumulation in shoots of *indica *rice independent from the effect of OsHMA3, another Cd transporter described in the next section.

### Xylem loading and root-to-shoot translocation

The ability of xylem-mediated Cd translocation into shoots is shown as a major determinant for shoot Cd accumulation in many plants including rice (Hart et al. 2006; Mori et al. 2009; Uraguchi et al. 2009b). In *A. thaliana *and *A. halleri *(a Cd/Zn hyperaccumulator), the key transporters for xylem Cd transport have been first identified. In *A. thaliana*, the P_1B_-type ATPase AtHMA2 and AtHMA4 regulate root-to-shoot translocation of Cd and Zn (Hussain et al. 2004; Verret et al. 2004; Wong and Cobbett 2009). In *A. halleri*, AhHMA4, a homolog of AtHMA4 plays a critical role in translocation of Cd and Zn into shoots (Hanikenne et al. 2008). The enhanced promoter activity and increased gene copy number of *AhHMA4 *result in higher expression of *AhHMA4 *in root stele and contribute to hyperaccmulation and hypertolerance of Cd and Zn in *A. halleri*.

Following the identification of the genes for xylem Cd transport in *A. thaliana *and *A. halleri*, OsHMA3 has been identified as a regulator for xylem Cd transport in rice by mediating vacuolar sequestration of Cd in root cells (Miyadate et al. 2011; Ueno et al. 2010). Compared to AtHMA4 and AhHMA4, OsHMA3 has some unique features. All these HMAs mediate Cd efflux transport, but OsHMA3 reportedly does not transport other metals such as Zn, whereas AtHMA4 and AhHMA4 functions in both Zn and Cd transport. Subcellular localization also differs between OsHMA3 and others. OsHMA3 is suggested to be localized to the vacuolar membrane, but AtHMA4 and AhHMA4 are localized to the plasma-membrane. The major difference of OsHMA3 and *Arabidopsis *HMA4s is the physiological function in plants. In the Nipponbare background, RNAi-mediated knock-down of *OsHMA3 *increased root-to-shoot Cd translocation and the overexpression reduced shoot Cd accumulation (Ueno et al. 2010). They suggests that in Nipponbare, OsHMA3 functions in vacuolar compartmentation of Cd in root cells and hence reduces the xylem loading of Cd and subsequent shoot Cd accumulation (Ueno et al., 2010), whereas AtHMA4 and AhHMA4 facilitate loading of Cd into the xylem. This finding reveals the mechanism for limiting Cd translocation into shoots in *japonica *rice.

At the same time, they found a single amino acid substitution in OsHMA3 from some *indica *cultivars (Anjana Dhan, Cho-ko-koku and Jarjan) which results in the loss-of-function (Miyadate et al. 2011; Ueno et al. 2011; Ueno et al. 2010). These cultivars show remarkably high Cd accumulation in shoots and grains even compared to other *indica *cultivars under the moderately Cd contaminated soil and the non-Cd-contamintation soil (Uraguchi et al. 2009b). Imaging analyses using PETIS demonstrated more rapid and greater Cd translocation from root-to-shoot in these Cd-high-accumulating cultivars (Ishikawa et al., 2011). The loss-of-functional allele of OsHMA3 is responsible for exceedingly higher root-to-shoot Cd translocation in these cultivars, because the non-functional OsHMA3 may result in failure of Cd compartmentation into vacuoles in root cells (Miyadate et al. 2011; Ueno et al. 2011; Ueno et al. 2010). This allele has not been detected in several *japonica *cultivars and also other *indica *cultivars such as Habataki (Takahashi et al. 2011), suggesting that this *OsHMA3 *is not the casual gene for general difference in Cd accumulation between *indica *and *japonica *rice.

### Phloem transport into grains

There has been no report of transporters for phloem Cd transport in plants so far, although in rice, phloem mediates nearly 100% of the Cd deposition into grains (Tanaka et al. 2007). Xylem-to-phloem Cd transfer at nodes is suggested (Fujimaki et al. 2010) and phloem Cd transport through a panicle neck shows genotypic variation (Kato et al. 2010). These suggest the existence and involvement of transporters at nodes for phloem Cd transport into grains. Remobilization of Cd from leaf blades to grains (Rodda et al. 2011) also appears to be mediated by phloem transport.

Recently, we have identified a transporter gene involved in phloem Cd transport (Uraguchi et al. 2011). This rice gene, named *OsLCT1*, is the homolog of wheat Low-affinity Cation Transporter1 (Clemens et al. 1998), and encodes a Cd-efflux transporter on the plasma-membrane. *OsLCT1 *expression was higher in leaf blades and nodes during reproductive stages. Especially in node I, the uppermost node, *OsLCT1 *was mainly expressed in diffuse vascular bundles which connected to panicles. In the Nipponbare background, Cd levels in grains and phloem exudate from leaf blades were substantially reduced in RNAi plants compared to control plants, although Cd concentration in xylem sap did not differ. These results suggest that OsLCT1 in leaf blades functions in Cd remobilization by phloem, and in node I, OsLCT1 is likely to play a part in intervascular Cd transfer from enlarged large vascular bundles to diffuse vascular bundles, which connect to the panicle. This is the first identification of a transporter for phloem Cd transport in plants.

### Other genes related to Cd accumulation and tolerance

In addition to the transporters involved in long-distance Cd transport processes described above, rice genes related to excess Cd stress have been reported. These genes might function in subcellular Cd transport such as exclusion of Cd from cytoplasm to apoplast and compartmentation into vacuoles as well as damage repairing processes that appear to be a mechanism underlying plant Cd tolerance (Clemens 2001; Uraguchi et al. 2009a). In this section, recent findings of rice genes possibly functioning in Cd homeostasis are reviewed. Although understanding of Cd tolerance may not directly contribute to advance establishment of "low-Cd-rice", the genes related to Cd tolerance may be potentially applied to manipulate rice Cd accumulation by regulating such as subcellular Cd transport.

Several ATP-binding cassette (ABC) proteins are suggested to mediate vacuolar compartmentation of Cd-glutathione and/or phytochelatin (PC) conjugates in baker's yeasts (Li et al. 1997), fission yeasts (Prévéral et al. 2009), the worm *Caenorhabditis elegans *(Vatamaniuk et al. 2005) and *A. thaliana *(Park et al. 2012). A member of ABC proteins of human (Tommasini et al. 1996) and *A. thaliana *(Kim et al. 2007) functions in Cd extrusion from the cytoplasm. We have recently reported that rice *OsPDR5/ABCG43 *is likely to encode an ABC-type protein for cellular Cd tolerance (Oda et al. 2011). The transcript of *ABCG43 *was remarkably induced in rice roots by Cd treatments, suggesting a possible involvement of ABCG43 in Cd tolerance in rice, although detailed mechanisms should be further investigated. A rice transporter for a Cd-PC conjugate would be also further identified, as demonstrated in *A. thaliana *(Park et al. 2012).

There are reports of rice genes for new mechanisms of Cd tolerance. A novel rice gene *Low cadmium *(*LCD*) is involved in Cd accumulation and tolerance (Shimo et al. 2011). Knock-out of *LCD *resulted in reduced Cd accumulation and increased growth under excess Cd. But LCD-GFP was localized to the cytoplasm and nucleus, suggesting that LCD is not a membrane-transporter. *LCD *is not homologous to any other genes, and the authors concluded that LCD is a novel protein related to Cd homeostasis. Kuramata et al. (2009) reported that a novel cysteine-rich peptides encoded by *OsCDT1 *is possibly involved in rice Cd tolerance. Overexpression of *OsCDT1 *in *A. thaliana *increased the growth of plants under Cd exposure (Kuramata et al. 2009).

**Figure 2:**
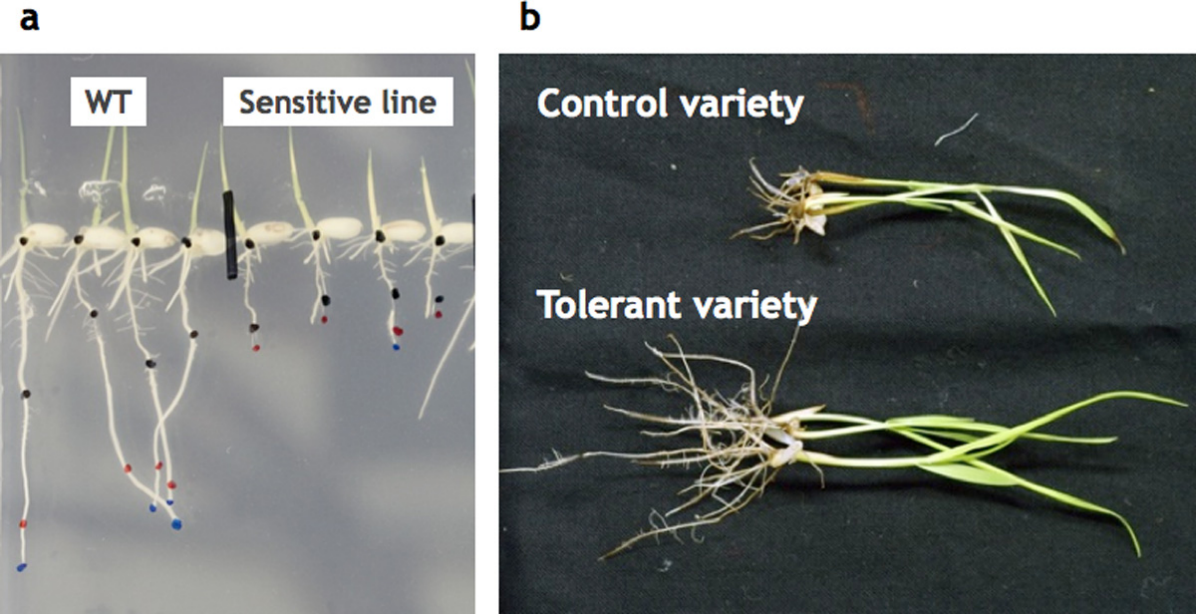
**Examples of a Cd-sensitive line (a) and a Cd-tolerant cultivar (b)**. These plants show different phenotypes under excess Cd treatments.

These reports indicate that novel mechanisms mediated by unknown molecules functions in Cd tolerance in plants and also indicate that rice is a potential genetic material for mining new genes related to Cd stress and/or accumulation. We have screened mutant populations and various genotypes and found several Cd-sensitive lines (Figure 2a) and one Cd-tolerant line (Figure 2b). Because there exist few examples of Cd-tolerant lines, analyses of these lines would contribute to advance understanding of Cd toxicity and tolerance in organisms.

### Approaches for reducing grain Cd accumulation

Considering substantial contribution of Cd in rice grains to higher Cd intake in Asian people, reducing Cd pollution of paddy fields and rice grain Cd accumulation are major issues to the field of agricultural science. Not only in the case of "Itai-itai disease" in Japan, but in some areas in China and Thailand, there are reports of Cd contamination of paddy soils and rice grains and renal disfunction in the populations (Nordberg et al., 1997; Jin et al., 2002; Honda et al., 2010). In Japan, the Ministry of Agriculture, Forestry, and Fisheries of Japan encourages rice growers to keep paddy fields flooded before and after heading to reduce Cd uptake by rice in Cd-contaminated areas. This is based on the fact that under the flooded condition, the soil is under a reductive condition and a large part of Cd in the soil forms CdS with low solubility (Iimura and Ito 1978). On the other hand, once the field is drained, the soil becomes an oxidative condition and CdS in the soil is changed to Cd^2+ ^which is much available to plants (Ito and Iimura 1976). This flooding water management before and after heading drastically reduces grain Cd concentrations, but on contrary, this treatment increases arsenic concentration in grains (Arao et al. 2009). Moreover, it seems difficult to uniform the flooding condition through entire paddy fields during the treatment, which may partially fail in reducing Cd availability in soils. Soil removal, replacement and soil inversion are possible other options and these have been conducted in some highly Cd-polluted paddy areas in Japan. This method is a quick solution for the contamination, but is cost-ineffective and requires a large amount of alternative soils. These features make it unpractical to apply this method to a much larger areas of paddies with moderate Cd contamination. Alternatively, several approaches to clean-up moderately Cd-polluted paddy soils have been suggested. Phytoextraction using the high-Cd-accumulating cultivar such as Cho-ko-koku (Murakami et al. 2009) and chemical washing of soils using ferric chloride (Makino et al. 2008) are possible methods in terms of efficiency, cost and environmental effects.

In combination to these soil remediation methods, establishment of "low-Cd-rice" based on genetic and molecular findings is expected as a promising approach to reduce human Cd intake. Regulation of Cd transporters by the transgenic technique has been used successfully to reduce Cd accumulation in rice plants. Overexpression of a functional allele of *OsHMA3 *in Nipponbare background resulted in drastic reduction of grain Cd concentration under a soil with relatively high Cd concentration (1.5 mg/kg) (Ueno et al. 2010). These *OsHMA3 *overexpressing rice reported in their manuscript did not show any reduction in zinc and iron contents in grains, although the growth of the plants was not reported. This phenotype is noteworthy because zinc and iron are important minerals for human. However, Cd accumulation of these *OsHMA3 *overexpressing rice under moderate or slight Cd contamination often observed in fields should be examined further.

Knock-down of *OsLCT1 *also reduced grain Cd by 50% under the soil containing 0.2 mg Cd/kg (Uraguchi et al. 2011). This soil Cd concentration is comparable to the average of Cd levels in Japanese soils, suggesting the significant potential of *OsLCT1 *alleles in establishing a rice cultivar which accumulates less Cd in grain under soils with slight Cd contamination. Importantly, the growth and mineral contents in grains were not negatively affected in these *OsLCT1*-knockdown plants.

These studies are good examples of the molecular approach based on physiological observations. Xylem loading and phloem transport are both shown as important transport processes determining grain Cd accumulation and the regulation of the responsible genes for the respective process enables reducing grain Cd levels without affecting quantity and quality of products. But one of the further issues for marketing "low-Cd-rice" in Japan is to establish "low-Cd-rice" by a non-transgenic approach. In Japan, the present consumers largely prefer non-transgenic agricultural products rather than those of genetically modified plants. Screening non-functional allele of *OsLCT1 *from chemically or radiation mutagenized populations with a background of major cultivars such as Koshihikari and establishing "low-Cd Koshihikari" is a possible practical approach. Another possible strategy is a marker-assisted breeding. For example, identification of a QTL for low-Cd-accumulation from a low-Cd-accumulating cultivar will enable to establish "low-Cd Koshihikari" by introducing the low-Cd QTL into Koshihikari by a marker-assisted breeding.

Manipulation of transporter genes expression also enables to generate high-Cd-accumulating rice. Overexpression of *OsNramp1 *(Takahashi et al. 2011) and *OsIRT1 *(Lee and An 2009) increased shoot Cd accumulation in rice plants. Knock-down of *OsHMA3 *also increased root-to-shoot Cd translocation (Ueno et al. 2010). These high-Cd-accumulating plants will be applied to remove Cd from Cd-polluted paddy soils (phytoextraction). Abe et al. (2011) introduced a non-functional allele of *OsHMA3 *from a high-Cd-accumulating cultivar Jarjan into Koshihikari by marker-assisted selection. These plants with a Jarjan allele of *OsHMA3 *showed a good Cd-removing ability from soils in a background of Koshihikari (Abe et al. 2011). They demonstrated that marker-assisted selection enabled to generate a high-Cd-accumulating rice with resistance to shattering and lodging and with larger biomass. These plants will increase the efficiency of phytoextraction operation in the field compared to using the high-Cd-accumulating rice such as Cho-ko-koku and Jarjan.

## Conclusions

Within the last few years, understanding of Cd transport and identification of the responsible genes in rice have been largely advanced. But it is possible that unknown transporters or other molecules are involved in rice Cd transport and tolerance. Moreover, mining of useful alleles is essential to establish non-transgenic low/high-Cd-accumulating rice. The progress of understanding and application in rice regarding Cd transport may be extended to other cereals.

## Competing interests

The authors declare that they have no competing interests.

## Authors' contributions

SU and TF wrote the manuscript. Both authors have read and approved the final manuscript.
